# Protein Abundance of Drug Transporters in Human Hepatitis C Livers

**DOI:** 10.3390/ijms23147947

**Published:** 2022-07-19

**Authors:** Marek Droździk, Joanna Lapczuk-Romanska, Christoph Wenzel, Łukasz Skalski, Sylwia Szeląg-Pieniek, Mariola Post, Marta Syczewska, Mateusz Kurzawski, Stefan Oswald

**Affiliations:** 1 Department of Pharmacology, Pomeranian Medical University, Powstancow Wlkp 72, 70-111 Szczecin, Poland; joanna.lapczuk.romanska@pum.edu.pl (J.L.-R.); lukasz.skalski@pum.edu.pl (Ł.S.); sylwia.szelag.pieniek@pum.edu.pl (S.S.-P.); mkurz@pum.edu.pl (M.K.); 2 Department of Pharmacology, Center of Drug Absorption and Transport, University Medicine Greifswald, 17489 Greifswald, Germany; christoph.wenzel@med.uni-greifswald.de; 3 Department of General and Transplantation Surgery, County Hospital, 71-455 Szczecin, Poland; mariolapost@wp.pl; 4 Department of Infectious Diseases, Hepatology and Liver Transplantation, Pomeranian Medical University, Arkonska 4, 71-455 Szczecin, Poland; martaws@wp.pl; 5 Institute of Pharmacology and Toxicology, Rostock University Medical Center, 18051 Rostock, Germany; stefan.oswald@med.uni-rostock.de

**Keywords:** hepatitis C, liver, cirrhosis, Child–Pugh score, drug transporter, protein quantification, real-time PCR, liquid chromatography-mass spectrometry

## Abstract

Transmembrane drug transport in hepatocytes is one of the major determinants of drug pharmacokinetics. In the present study, ABC transporters (P-gp, MRP1, MRP2, MRP3, MRP4, BCRP, and BSEP) and SLC transporters (MCT1, NTCP, OAT2, OATP1B1, OATP1B3, OATP2B1, OCT1, and OCT3) were quantified for protein abundance (LC-MS/MS) and mRNA levels (qRT-PCR) in hepatitis C virus (HCV)-infected liver samples from the Child–Pugh class A (*n* = 30), B (*n* = 21), and C (*n* = 7) patients. Protein levels of BSEP, MRP3, MCT1, OAT2, OATP1B3, and OCT3 were not significantly affected by HCV infection. P-gp, MRP1, BCRP, and OATP1B3 protein abundances were upregulated, whereas those of MRP2, MRP4, NTCP, OATP2B1, and OCT1 were downregulated in all HCV samples. The observed changes started to be seen in the Child–Pugh class A livers, i.e., upregulation of P-gp and MRP1 and downregulation of MRP2, MRP4, BCRP, and OATP1B3. In the case of NTCP, OATP2B1, and OCT1, a decrease in the protein levels was observed in the class B livers. In the class C livers, no other changes were noted than those in the class A and B patients. The results of the study demonstrate that drug transporter protein abundances are affected by the functional state of the liver in hepatitis C patients.

## 1. Introduction

Hepatitis C virus (HCV) is an RNA virus, which infects hepatocytes, leading to liver dysfunction ending in the natural course of the disease in cirrhosis and/or hepatocellular carcinoma. Recently, the disease prognosis has been markedly improved because of the introduction of new drugs directly targeting virus structures by direct-acting antiviral (DAA) drugs, which enable not only disease control, but also HCV eradication [1,2]. Clinical efficacy of DAA agents depends in part on drug-metabolizing enzyme actions localized mainly in hepatocytes, and on intracellular drugs and their metabolites trafficking regulated by drug carriers and transporters [3]. 

Hepatocytes are endowed by both uptake and efflux transporter systems handling drug molecules. The most clinically relevant drug transporters and carriers defined in human hepatocytes belong to efflux transporters localized in the apical (canalicular) membrane, i.e., P-glycoprotein (P-gp and ABCB1), bile-salt export pump (BSEP and ABCB11), breast cancer resistance protein (BCRP and ABCG2), and multiple drug-resistance-associated protein 2 (MRP2 and ABCC2), as well as multidrug and toxin extrusion protein 1 (MATE1 and SLC47A1). In the basolateral (sinusoidal) membrane function, uptake carriers are from OATP family, i.e., organic anion transporting polypeptide 1B1 (OATP1B1 and SLCO1B1), OATP1B3 (SLCO1B3), and OATP2B1 (SLCO2B1), as well as SLC22A family, i.e., organic cation transporter 1 (OCT1 and SLC22A1) and organic anion transporter OAT2 (SLC22A7). The basolateral membrane is also endowed with efflux transporters, such as MRP3 (ABCC3), MRP4 (ABCC4), and MRP6 (ABCC6) [4]. 

These carriers and transporters are also involved in the regulation of transmembrane shuttle of DAAs, and thus contribute to their metabolism and elimination. In this regard, it was demonstrated that OATP1B1 and OATP1B3 mediate the hepatic uptake of glecaprevir, grazoprevir, voxilaprevir, daclatasvir, pibrentasvir, and velpatasvir, as well as OATP2B1 mediating that of voxilaprevir and velpatasvir [5]. The biliary efflux transporters are involved in the transport of many DDAs, namely P-gp and BCRP transporting glecaprevir, grazoprevir, voxilaprevir, daclatasvir, elbasvir, ledipasvir, pibrentasvir, velpatasvir, and sofosbuvir [5]. In addition, the major intestinal transporters, i.e., P-gp, BCRP, MRP1, MRP2, and MRP3, are also important determinants of their oral bioavailability [6]. 

Drug transporters and carriers also participate in trafficking of many other drugs than DAAs drugs used in patients affected by HCV infection. Drug labels of many clinically used agents state that, due to unavailability of pharmacokinetic data and/or dosing information in liver failure patients, administration of drugs is not recommended or contraindicated. On the other hand, ethical concerns virtually preclude methodologically rigorous pharmacokinetic or drug–drug interaction (DDI) studies in advanced stages of liver dysfunction, especially for medicines of nontherapeutic value. Proteomic studies, which provide quantitative information on hepatic drug transporters in HCV patients with different stages of the disease, provide data that enable stratification of potential risks derived from altered pharmacokinetics (and possibly pharmacodynamics) of administered drugs and prediction of oral drug bioavailability and DDIs using physiologically based pharmacokinetic (PBPK) modeling and simulation.

The existing information suggests that hepatitis C virus infection induced changes in hepatocytes, resulting in deregulation of membrane transporters. Billington et al. [7] showed decreased protein levels of sodium-dependent taurocholate co-transporting polypeptide (NTCP), OATP1B3, OCT1, bile salt export pump (BSEP), and MRP2, with no change in P-gp, MRP3, OATP1B1, or OATP2B1 in HCV-induced liver cirrhosis. However, this study did not define stages of liver function of the included samples. In addition, Wang et al. [8] found an increase in MATE1 but decrease in BSEP, MRP2, NTCP, OATP1B3, OCT1, and P-gp, or no change in BCRP, MRP3, OATP1B1, and OATP2B1. A decrease in the protein abundance of MRP2 and OATP2B1 in hepatitis C cancer livers (in some samples, overlapped with alcoholic liver disease and nonalcoholic fatty liver disease) was also reported [9]. Our preliminary data in HCV livers mostly corroborate the abovementioned observations (decrease in BSEP, OCT1, OATP1B1, and OATP2B1 and stable levels of P-gp, MRP2, MRP3, MRP4, BCRP, NTCP, OCT3, OAT2, and OATP1B3 transporter proteins). This initial study also suggested disease and liver function stage-dependent changes. Rather similar changes were observed in HCV and alcoholic livers, as well as primary biliary cholangitis and primary sclerosing cholangitis, i.e., upregulation of protein levels of efflux pumps in cholestatic and reduced uptake transporter abundance in parenchymal hepatic damage [10].

In the present study, we were able to expand the number of the studied hepatitis C samples, which allows the provision of not only quantitative proteomic (liquid chromatography-tandem mass spectrometry, LC-MS/MS) data on drug transporter status in HCV, but also to stratify expression levels in different stages (according to the Child–Pugh classification) of HCV-induced liver dysfunction.

## 2. Results

### 2.1. mRNA Expression

**Figure 1:**
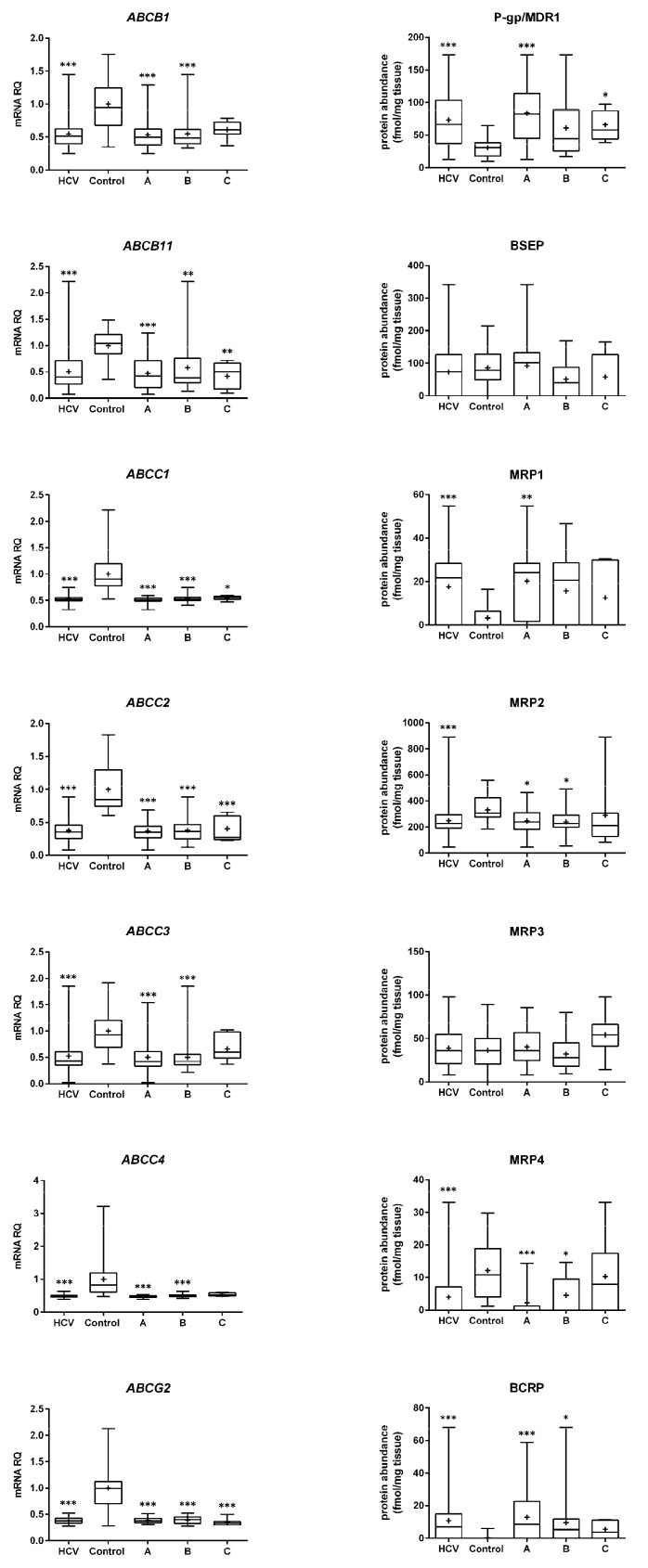
Gene expression (**left**) and protein abundance (**right**) of ABC transporters in livers from hepatitis C (HCV, *n* = 58) patients, also subdivided according to the Child–Pugh score into stages: A (*n* = 30), B (*n* = 21) and C (*n* = 7), presented in boxes on the right side to the control livers (*n* = 20). The data are represented as box plots of the median (horizontal line), 75th (top of box), and 25th (bottom of box) quartiles; the smallest and largest values (whiskers) and mean (+) are shown. mRNA level of the analyzed genes was expressed as relative amounts to the mean value for the control group (ΔΔCT method). Statistically significant differences: * *p* < 0.05, ** *p* < 0.01, *** *p* < 0.001 (Kruskal–Wallis test or Mann–Whitney U test) in comparison to the controls.

mRNA expression of the studied ABC and SLC transporters was affected by HCV infection and the liver functional state. Statistical analysis demonstrated more prominent changes in mRNA expression than in protein abundance. In the case of ABC transporters, expression of all genes was significantly downregulated in HCV patients in all disease stages (except for *ABCB1*, *ABCC3*, and *ABCC4* in the Child–Pugh class C) (Figure 1). 

**Figure 2:**
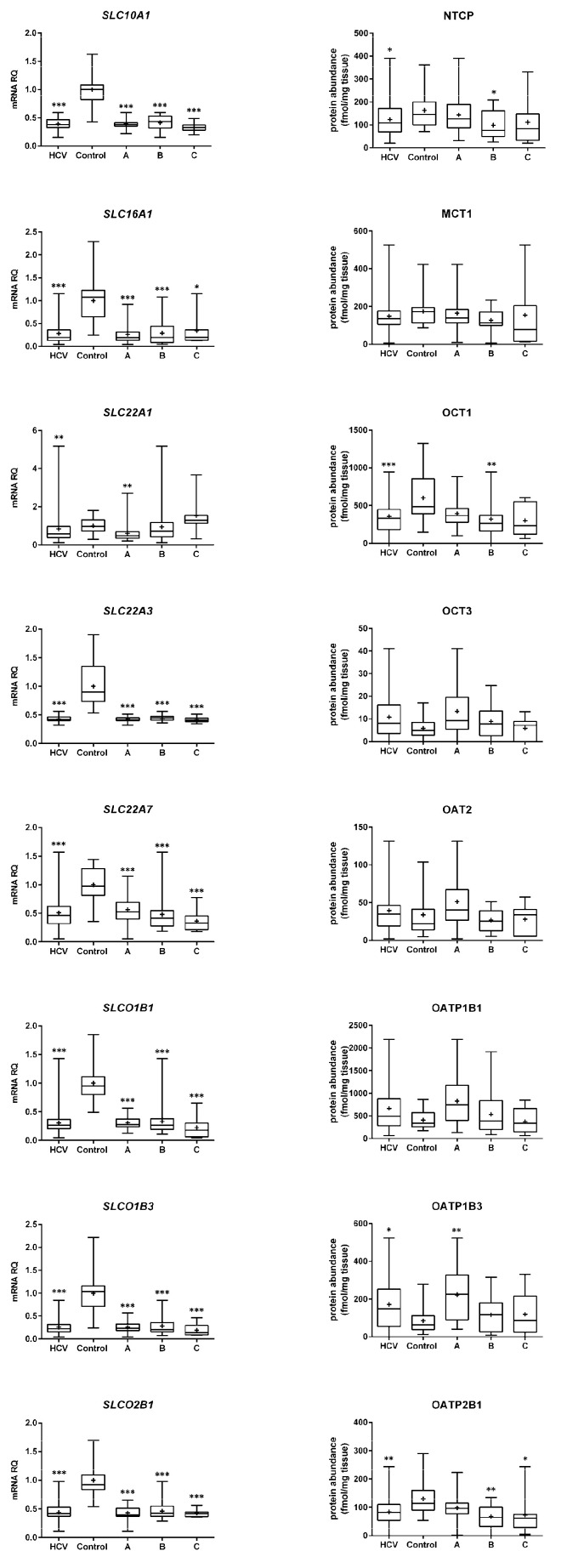
Gene expression (**left**) and protein abundance (**right**) of SLC transporters in livers from hepatitis C (HCV, *n* = 58) patients, also subdivided according to the Child–Pugh score into stages: A (*n* = 30), B (*n* = 21), and C (*n* = 7), presented in boxes on the right side to the control livers (*n* = 20). The data are represented as box plots of the median (horizontal line), 75th (top of box), and 25th (bottom of box) quartiles; the smallest and largest values (whiskers) and mean (+) are shown. mRNA level of the analyzed genes was expressed as relative amounts to the mean value for the control group (ΔΔCT method). Statistically significant differences: * *p* < 0.05, ** *p* < 0.01, *** *p* < 0.001 (Kruskal–Wallis test or Mann–Whitney U test) in comparison to the controls.

Similar to ABC transporters, the SLC carriers gene expressions were also reduced in all the Child–Pugh class livers, with the exception of SLC22A1 (Figure 2). 

### 2.2. Protein Abundance

Protein levels of several studied transporters were not significantly affected by HCV infection, and protein abundance was stable irrespective of the disease stage, i.e., BSEP, MRP3, MCT1, OAT2, OATP1B3, and OCT3 (but a downregulation trend can be noted in OATP1B1 and OCT3). In contrast, the protein levels of P-gp, MRP1, BCRP, and OATP1B3 were upregulated, whereas those of MRP2, MRP4, NTCP, OATP2B1, and OCT1 were downregulated in all HCV samples. The observed changes started to be seen in the Child–Pugh class A livers, i.e., upregulation of P-gp and MRP1 and downregulation of MRP2, MRP4, BCRP, and OATP1B3. In the case of NTCP, OATP2B1, and OCT1, decrease in the protein levels was evidenced in class B livers (Figure 1 and Figure 2). In the class C livers, no other changes were noted than those in the class A and B patients. 

**Figure 3:**
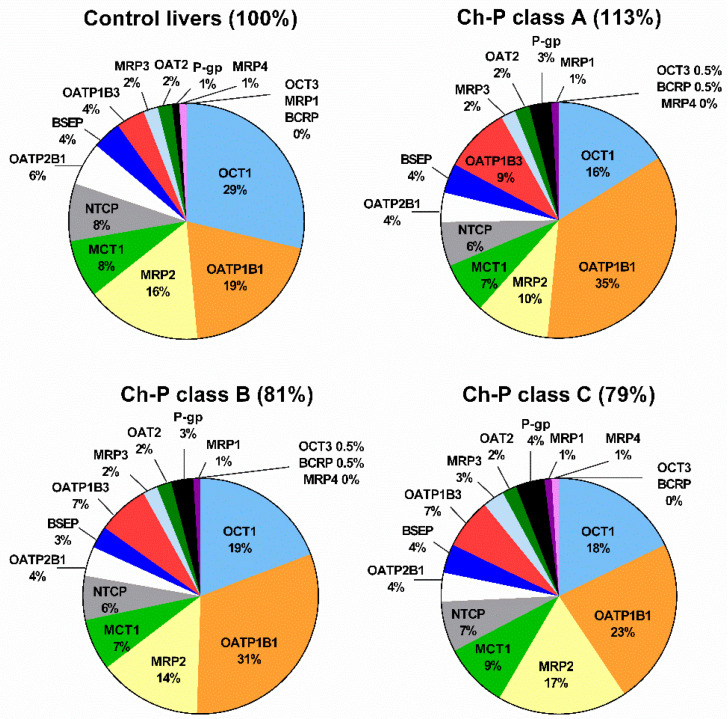
The pie chart of the individual drug transporter proteins in the control livers, as well as in hepatitis C livers stratified according to Child–Pugh score (Ch-P class A, B, or C). The pie charts show the abundance of each transporter protein as a percentage of the sum of all transporter proteins’ abundance. Percentages in brackets indicate a total transporter protein abundance in comparison to that in the control livers (indicated as 100%).

In the control livers, the most abundant transporter identified was OCT1 (29%), which was followed by OATP1B1 (19%), MRP2 (16%), MCT1 (8%), and NTCP (8%). Infection by hepatitis C virus resulted in changes in the protein levels of the transporters. In livers of all stages (according to the Child–Pugh classification), the most abundant transporters were OATP1B1, and then OCT1, MRP2, MCT1, and NTCP (Figure 3).

The correlation analysis between the respective gene transporters’ expression and protein abundance did not reveal strong (r > 0.6) correlations, both in the control samples as well as in the hepatitis C livers, except for positive correlation of *SLCO1B3*/OATP1B3, as well as negative of *ABCG2*/BCRP in the Child–Pugh class C livers (Table 1). 

## 3. Discussion

Information about proteomic status of drug/membrane transporters and carriers in the liver in its healthy and disease states gives insights into liver pathologies themselves, as well as on drug pharmacokinetics and, thus, therapeutic responses and potential side effects in patients with liver dysfunction. As the correlation between mRNA and protein levels is not always satisfactory, the reliable protein quantification provides better prediction parameters than gene expression data. Our previous findings, which included various liver pathologies (hepatic-virus-induced liver damage, alcoholic liver disease, autoimmune hepatitis, primary biliary cholangitis, and primary sclerosing cholangitis), demonstrated a dissociation between gene expressions and protein abundances of drug transporters and carriers [10]. 

So far, only limited information about proteomic status of the transporters is available, especially in different functional stages of the liver. The abovementioned study demonstrated that different liver pathologies affected various ABC and SLC transporters [10]. The HCV samples of the aforementioned study (also included in the current analysis, 21 samples; Child–Pugh class: A = 7, B = 10, and C = 4) revealed that the disease was associated with significant downregulation of BSEP, OCT1, OATP1B1, and OATP2B1 protein abundances and stable levels of P-gp, MRP1, MRP2, MRP3, MRP4, BCRP, NTCP, OCT3, OAT2, and OATP1B3. Those results mostly corroborate with the findings of the present study, but analysis of a larger number of cases confirmed downregulation of OATP2B1 and OCT1, and, additionally, of MRP2, MRP4, and NTCP. In the previous study, any upregulation of the transporters was seen in HCV samples, whereas the present and more comprehensive analysis showed decreased levels of P-gp, MRP1, BCRP, and OATP1B3. Similar to our present observations, Wang et al. [8] noted a decrease in MRP2, NTCP, and OCT1, as well as no change in MRP3 and OATP1B1. Contrary to the increase reported in the present study, Wang et al. [8] reported decreased protein levels of OATP1B3 and P-gp. This study also revealed a downregulation of BCRP and OATP2B1 not observed by Wang et al. (no change). Decreased BSEP levels in the report of Wang et al. [8] were different from the stable abundance of the transporter protein in the present study. Mostly similar results were reported by Billington et al. [7] in chronic hepatitis C infection (but not stratified according to the liver functional state). An analysis of a small number of hepatitis C livers was also reported by El-Khateeb et al. [9], who revealed downregulation of MRP2 and OATP2B1 proteins. A summary of the available proteomic information about drug transporters and carriers in hepatitis C is presented in Table 2. Analysis of transporter abundance of all studies with hepatitis C patients suggests downregulation of NTCP, BSEP, and OCT1 uptake carriers and MRP2 efflux transporter, as well as stable levels of MRP3, BCRP, and OATP1B1. However, most of these studies did not provide detailed information about patient stratification according to the Child–Pugh classification, and only El-Khateeb et al. [9] specified that the analysis included five patients from the Child–Pugh class A and four subjects from class B (without detailed protein abundance results).

The differences in transporter protein abundance in the abovementioned studies, apart from methodological aspects (e.g., Billington et al. [7] and Wang et al. [8] used a membrane enrichment kit but not whole liver tissue), may also stem from analysis of samples harvested from subjects of various Child–Pugh classes. Progression of hepatitis-C-induced functional and structural changes produces a bias of sampling, with a risk of non-hepatocyte (parenchymal) tissue harvesting. The fraction of functional liver mass was shown to be 69%, 55%, and 28% of the healthy liver in the Child–Pugh class A, B, and C, respectively [12]. In the present study, an effort was undertaken to collect macroscopically unaffected liver samples. However, reduction in a number of hepatocytes is paralleled with the expansion of nonparenchymal cells, e.g., sinusoidal endothelial cells, Kupffer cells, and hepatic stellate cells, as well as fibroblasts and transdifferentiated myofibroblasts and fibrous connective tissue [13] and, thus, might affect study findings. 

The present study demonstrates for the first time quantitative data on drug transporter protein abundance in different liver functional stages in samples from one organ disease, i.e., hepatitis C. The findings reveal that changes in the transporter protein abundances are associated with the functional state of the liver. The Child–Pugh class A livers are characterized by significant protein abundance upregulation of P-gp and MRP1 and downregulation of MRP2, MRP4, BCRP, and OATP1B3. Progression of hepatitis C infection to class B triggered changes in NTCP, OATP2B1, and OCT1, the levels of which decreased. These results suggest that the most dynamic changes in the transporter abundance in the liver are observed in the early stages of hepatitis C virus infection, and no new, not observed in class A and B livers, significant effects in transporter levels were seen in the Child–Pugh class C specimens. However, in this class, a relatively small number of samples was analyzed, which might affect results of the statistical analysis. 

The changes in the transporters’ expression/abundance can be, in part, ascribed to altered cytokine status produced by hepatitis C virus infection. Hepatitis C is an inflammatory disease associated with elevated expression levels of IL-6 and TNF-α (tumor necrosis factor-α) [14]. It is also documented that liver-infiltrating T cells from chronic hepatitis C patients produce IFN-γ [15], apoptotic hepatocytes release IL-1α [16], and macrophages exposed to HCV induce IL-1β and IL-18 secretion [17]. These cytokines were demonstrated to be involved in the transcriptional regulation of some drug transporters and may explain the expression/abundance changes. TNF-α can downregulate ABCC2/MRP2 (by reducing the activity of CAR and PXR) levels [18]. Downregulation of NTCP and OATP2B1 was observed in hepatocytes (precision-cut human liver slices) exposed to IFN-γ, TNF-α, or IL-6 [19]. The downregulation of ABCC4/MRP4 may result from nuclear factor erythroid-2-related factor-2 (Nrf2) responses to oxidative liver injury, as the transcription factor was shown to be involved in the regulation of human ABCC4 transcription [20]. However, not all findings of the present study are in keeping with in vitro and ex vivo conducted experiments [21]. 

The correlation analysis did not reveal strong correlations between the studied gene expressions and the respective protein abundances irrespective of the functional state of the organ. These findings are in keeping with other published data [22,23,24] and strongly suggest that transporter protein levels mostly do not correlate with mRNA expression. Therefore, findings based only on transcriptome analysis do not reflect real changes in transporter abundance. 

The finding of the present study can only partially explain pharmacokinetic characteristics of HCV recommended drugs. Velpatasvir is a substrate of P-gp, BCRP, and OATP1B1/1B3 (some 23% of dose metabolized by CYP3A4, 2C8, and 2B6, with minimal renal elimination and some biliary excretion), and its AUC levels (when co-administered with sofosbuvir) increase stepwise in mild (by 9.2%) and moderate (29.9%) HCV-induced hepatic impairment. Co-administration of cyclosporine, a known inhibitor of uptake carrier and efflux transporters, including OATPs, P-gp, MRP2, and BCRP [25], resulted in AUC increase in the drug by ~two-fold (summarized in [26]). The studies in hepatic impairment (accessed by Child–Pugh score) in HCV-negative patients with liver cirrhosis, hepatitis B infection, alcoholic liver disease, or previous HCV infection demonstrated an AUC increase in glecaprevir (a substrate of P-gp, BCRP, and OATP1B1/1B3, with about 28% of dose metabolized mostly by CYP3A4, minimal renal and some biliary excretion) (when co-medicated with pibrentasvir) in mild (by 33%), moderate (by 100%), and severe (1010%) hepatic impairment. Administration of a single 100 mg and single 400 mg cyclosporine dose increased the drug AUC by 37% and five-fold, respectively (summarized in [26]). In the same type of patients as in the case of glecaprevir, pharmacokinetics of pibrentasvir (a substrate of P-gp and BCRP; not metabolized, with some biliary elimination; 96.6% of radiolabeled drug recovered in feces with negligible metabolism observed and no measurable radioactivity in urine) was evaluated. An increase in AUC by 51%, 31%, and 415% was observed in Child–Pugh class A, B, and C patients, respectively. Co-medication with cyclosporine increased AUC of the drug by 1.9-fold. It should be stated that the proteomic quantitative results of the present study do not reflect the activity of the respective transporter proteins. All of the drugs cited above, i.e., glecaprevir, velpatasvir, and pibrentasvir, are not only transporter substrates, but also inhibitors of P-gp, BCRP, and OATP1B1/3 [27], which may determine final impact on drug molecule handling produced by the transporters. This complex situation should be addressed in future studies as intrahepatic drug concentrations are crucial for clinical efficacy of agents used in the treatment of HCV infection. Various impacts of different liver pathologies (recruited for glecaprevir and pibrentasvir study) on the transporter levels and presumably drug pharmacokinetics might also contribute to the observed discrepancies [10].

The current study findings suggest (proteomic quantitative data) that the downregulation of OCT1 and OATP2B1 may reduce hepatic uptake and efficacy of drugs operating in hepatocytes (despite potentially higher drug serum levels). Similar to lower abundance of hepatic uptake carriers, higher biliary efflux produced by upregulated P-gp or BCRP levels may involve an accelerated elimination of drugs from the liver. In contrast, OATP1B1/3 substrates (due to upregulated levels in HCV) may show higher hepatic accumulation and efficacy. 

Apart from pharmacokinetic aspects, where the findings of the study can be applied, the present observations may also help to explain some clinical features, i.e., a downregulation of NTCP noted in HCV subjects can contribute to a less common HCV patient superinfection rate with HBV than HBV patient superinfection with HCV (NTCP is a defined entry factor for HBV) [28]. 

It would also be interesting to combine the observed changes in membrane transporter levels with protein species involved with HCV infection in order to better define the observed interactions. In this framework, fluorescence assays [29,30] to directly monitor the evolution of HCV disease at the cellular level, atomic force microscopy to enable the acquisition of morphological information at the single molecule level [31], which can be extendable to HCV proteases [32], docking computational approaches to find flavivirus protease inhibitors [33,34], or integrative methodologies combining X-ray crystallography and cross-linking mass spectrometry studies [35] are some suitable examples to address this gap in information.

The protocol of the present study may produce some limitations. The number of the cases analyzed, especially in the Ch–P class C, is limited and reflects criteria for liver transplantation (i.e., patients are transplanted in a less advanced stage of the disease). However, it is the largest proteomic study on drug transporters in hepatitis-C-affected livers. The HCV liver samples were collected in the years 2007–2019 and treatment standards for the disease were modified, which might also affect the results, but we were able to exclude all reported co-morbidities. In this study, and in other published reports dissected liver samples were analyzed and, in more advanced stages of the disease (cirrhotic livers), sampling of non-hepatic parenchyma could impact the results. To reduce the sampling bias, efforts were made to dissect only the parenchymal liver tissue to analyze as many tissues rich in hepatocytes as possible. 

The present study results providing the transporter protein abundance values in HCV livers from the Child–Pugh class A to C can be used to better scale PBPK models of drugs predicted to be applied in patients suffering from HCV infection, especially of those not being inhibitors of the transporters and carriers. Pharmacokinetics of most of the clinically used drugs, including several DDA, were not evaluated in HCV patients with advanced liver disease (especially in the Child–Pugh class C subjects), with resulting lack of dosing information and/or contraindications to use [5]. Therefore, more detailed protein abundance information of drug transporters in all Child–Pugh stages in hepatitis C patients could increase the safety of clinical pharmacokinetics studies, with possible implementation of new treatment strategies in more advanced stages of the disease. 

## 4. Materials and Methods

### 4.1. Liver Samples

The control samples were harvested from metastatic livers from a site at least 5 cm distant from the tumor site. The tissues were collected from Caucasian patients diagnosed with metastatic colon cancer. The collected tissues did not show any pathological signs, as confirmed by histological examination (the samples were used as the controls in the previously published study [10]).

Hepatitis C (HCV) (diagnosed according to the standard clinical criteria) liver parenchymal tissue samples were dissected from the patients requiring liver transplantation. The liver tissue specimens were harvested during elective liver transplantation from the organ immediately after excision. The stage of liver dysfunction was classified according to the Child–Pugh score. Characteristics of the subjects are presented in Table 3. 

Tissue biopsies were taken from livers (control and pathological) under standard general anesthesia not later than 15 min after blood flow arrest. To reduce the sampling bias, efforts were made to dissect only the parenchymal liver tissue to analyze as many tissues rich in hepatocytes as possible. However, the contribution of other cellular and noncellular components might affect the study results, apart from the underlying liver pathology and disease stage. The liver samples were immediately snap frozen in liquid nitrogen for protein analysis or immersed in RNAlater (Applied Biosystems, Darmstadt, Germany) for RNA analysis, and then stored at −80 °C. The study protocol was approved by Bioethics Committee of the Pomeranian Medical University.

### 4.2. mRNA Isolation and Quantitative Real-Time RT-PCR

Total RNA was isolated from 25 mg of each tissue sample using Direct-zol RNA MiniPrep kit (Zymo Research, Irvine, CA, USA). RNA concentration and purity was assessed using DS-11 FX spectrophotometer (Denovix, Wilmington, DE, USA). cDNA was prepared using SuperScript^®^ VILO™ cDNA Synthesis Kit (Thermo Fisher Scientific, Waltham, MA, USA), with 500 ng of total RNA for 20 µL of reaction volume according to the manufacturer’s procedure. The gene expression levels were examined in duplicate using TaqMan Fast Advanced Master Mix and prevalidated TaqMan assays ABCB1 (Hs00184500_m1), ABCC1 (Hs01561502_m1), ABCC2 (Hs00166123_m1), ABCC3 (Hs00978473_m1), ABCC4 (Hs00988717_m1), ABCG2 (Hs01053790_m1), ABCB11 (Hs00184824_m1), SLC10A1 (Hs00161820_m1), SLC16A1 (Hs01560299_m1), SLC22A1 (Hs00427552_m1), SLC22A3 (Hs00222691_m1), SLC22A7 (Hs00198527_m1), SLCO1B1 (Hs00272374_m1), and SLCO1B3 (Hs00251986_m1), SLCO2B1 (Hs01030353_m1) in ViiA 7 Real-Time PCR System (Life Technologies, Carlsbad, CA, USA). Threshold values for each gene were set manually and mean cycles of threshold (CT) values were recorded. All CT values for each sample were normalized to the geometric mean value obtained for five housekeeping genes: GUSB (Hs00939627_m1), HMBS (Hs00609297_m1), PPIA (Hs04194521_s1), RPLP0 (Hs99999902_m1), and RPS9 (Hs02339424_g). Relative expression (relative quantity—RQ) of the analyzed genes was calculated by the ∆∆CT method—normalized to the mean value for the control group. 

### 4.3. Protein Quantification by Liquid Chromatography-Tandem Mass Spectrometry (LC−MC/MS)

Tissues placed in liquid nitrogen were mechanically disrupted in a stainless-steel mortar system. Approximately 40 mg tissue powder of each sample was lysed with 1 mL of 0.2% SDS and 5 mM EDTA containing 5 µL/mL Protease Inhibitor Cocktail Set III (Merck, Darmstadt, Germany) for 30 min at 4 °C on a platform shaker with 40 rpm (Polymax 1040, Heidolph, Schwabach, Germany). Total protein content of the whole tissue lysates was determined by bicinchinonic acid assay (Thermo Fisher) and 100 µg of each sample was processed using filter-aided sample preparation (FASP) [36]. Protein quantification of ABC transporters (P-gp, MRP1, MRP2, MRP3, MRP4, BCRP, and BSEP) and SLC transporters (MCT1, NTCP, OAT2, OATP1B1, OATP1B3, OATP2B1, OCT1, and OCT3) were measured by mass-spectrometry-based targeted proteomics using a validated LC−MS/MS method [37]. With the exception of P-gp, MRP4, BSEP, MCT1, NTCP, OAT2, and OCT3, all remaining proteins were analyzed by using two proteospecific peptides. In each case, one peptide was used for quantification, whereas the other served as a qualifier verifying the presence of the specific protein. For all peptides and their isotope-labeled internal standards, three mass transitions were used, respectively. The calculated protein values represent the mean of at least 2–3 mass transitions/peptide. The details of the procedure, peptides used, and mass transitions were published previously [10]. 

### 4.4. Statistical Analysis

The normality of the data (mRNA and protein abundance) was assessed by Shapiro–Wilk test. Due to a significant deviation from normal distribution, differences between study groups were evaluated using the nonparametric Mann–Whitney U test (control vs. HCV) and Kruskal–Wallis test for multiple comparisons with post hoc Dunn’s test (control vs. Child–Pugh class A, B, and C). The correlations were calculated with the Spearman rank test. *p* values of <0.05 were considered as significant. All of the statistical calculations were performed using Statistica 13.3 Software Package (TIBCO Software Inc., Palo Alto, CA, USA).

## 5. Conclusions

In the present paper, effects on HCV infection and the disease course on protein expression was documented. The findings demonstrate that the disease progression from mild to severe, i.e., from the Child–Pugh (Ch–P) score A to C, entails changes in transporter abundance. The observed changes started as early as in the Child–Pugh class A livers, i.e., upregulation of P-gp and MRP1 and downregulation of MRP2, MRP4, BCRP, and OATP1B3. In the Ch–P class B livers a decrease in the protein levels of NTCP, OATP2B1, and OCT1 was observed. In the Ch–P class C livers, the previously defined changes were preserved. However, not all studied transporters were affected by the disease, i.e., protein levels of BSEP, MRP3, MCT1, OAT2, OATP1B3, and OCT3 were not significantly changed in the course of HCV infection. So, the results of the study demonstrate that drug transporter protein abundances are affected by the functional state of the liver in hepatitis C patients. The new information presented in the study can be implemented in the construction of more adequate physiologically based pharmacokinetic (PBPK) models and could contribute to understanding of HCV infection pathophysiology. 

## Figures and Tables

**Table 1:** Correlation (Spearman coefficient, r) between protein and mRNA level of the ABC and SLC transporters in HCV cirrhotic livers (HCV) and disease stages (Child–Pugh class A, B, and C), as well as in the control.

*Gene*/Protein	Control *n* = 20	HCV *n* = 58	Child–Pugh Class A *n* = 30	Child–Pugh Class B *n* = 21	Child–Pugh Class C *n* = 7
*ABCB1*/P-gp	0.432	−0.251	−0.174	−0.270	−0.393
*ABCC1*/MRP1	−0.481 *	0.168	0.157	0.291	−0.079
*ABCC2*/MRP2	0.206	0.375 **	0.326	0.331	0.607
*ABCC3*/MRP3	0.029	0.264 *	0.304	0.092	0.571
*ABCC4*/MRP4	−0.220	0.304 *	0.430 *	0.041	−0.185
*ABCG2*/BCRP	LLOQ	−0.322*	0.012	−0.578 **	−0.811 *
*ABCB11*/BSEP	0.021	0.011	0.063	−0.069	0.197
*SLC10A1*/NTCP	0.066	0.268 *	0.189	0.357	0.321
*SLC22A1*/OCT1	0.424	0.265 *	0.195	0.104	0.714
*SLC22A3*/OCT3	0.296	−0.228	−0.289	−0.281	0.126
*SLC22A7*/OAT2	0.302	0.412 **	0.270	0.325	0.214
*SLCO1B1*/OATP1B1	0.295	−0.030	−0.071	−0.392	0.179
*SLCO1B3*/OATP1B3	0.519 *	0.025	−0.174	−0.234	0.893 **
*SLCO2B1*/OATP2B1	−0.077	0.191	−0.086	0.436 *	0.214

* *p* < 0.05; ** *p* < 0.01; LLOQ—lower limit of quantification.

**Table 2:** Protein abundance of hepatobiliary transporters in the available studies on hepatitis C livers (not stratified according to the Child–Pugh classification) in the whole liver tissue.

	This Study *n* = 58	Billington et al. [7] ^ *n* = 30	Wang et al. [8] *n* = 30	El-Khateeb et al. [9] ^#^ *n* = 9	Drozdzik et al. [10] *n* = 21
P-gp	↑	↔	↓		↔
MRP1	↑				↔
MRP2	↓	↓	↓	↓	↔
MRP3	↔	↔	↔	↔	↔
MRP4	↓				↔
BCRP	↑		↔	↔	↔
BSEP	↔	↓	↓		↓
NTCP	↓	↓	↓		↔
MCT1	↔				↔ *
OCT1	↓	↓	↓		↓
OCT3	↔				↔
OAT2	↔			↔	↔
OATP1B1	↔	↔	↔		↓
OATP1B3	↑	↓	↓		↔
OATP2B1	↓	↔	↔	↓	↓

*—studied in the same set of samples published in Drozdzik et al. [11]; ^—HCV-cirrhotic livers; ^#^—HCV and HCV + alcoholic liver disease or hepatocellular carcinoma or nonalcoholic fatty liver disease; ↑—increase; ↓—decrease; ↔—not changed.

**Table 3:** Characteristics of the subjects (mean ± SD). HCV—hepatitis C; Ch–P: A, B, C—Child–Pugh Class A, B, C; INR—International Normalized Ratio.

Parameter/Disease	Population Normal	Control *n* = 20	HCV *n* = 58	Ch-P A *n* = 30	Ch-P B *n* = 21	Ch-P C *n* = 7
Sex (male/female)		11/9	30/28	16/14	11/10	3/4
Age (years)		63 ± 10	56 ± 7	57 ± 7	55 ± 8	52 ± 9
Total bilirubin (mg/dL)	0.1–1.2	0.59 ± 0.25	1.75 ± 1.26	1.03 ± 0.57	2.05 ± 0.84	3.62 ± 1.78
Albumin (g/dL)	3.4–5.4	3.89 ± 0.38	3.38 ± 0.57	3.67 ± 0.49	3.23 ± 0.45	2.71 ± 0.40
INR	0.9–1.1	1.14 ± 0.21	1.30 ± 0.28	1.20 ± 0.22	1.29 ± 0.17	1.71 ± 0.36

## Data Availability

The data are deposited in the repository of Pomeranian Medical University in Szczecin: https://ppm.pum.edu.pl.
